# Complete Genome Sequence of *Escherichia coli* ABWA45, an *rmtB*-Encoding Wastewater Isolate

**DOI:** 10.1128/genomeA.00844-17

**Published:** 2017-08-24

**Authors:** Katrin Zurfluh, Roger Stephan, Jochen Klumpp, Magdalena Nüesch-Inderbinen, Jörg Hummerjohann, Claudia Bagutti, Roger Marti

**Affiliations:** a Institute for Food Safety and Hygiene, Vetsuisse Faculty University of Zurich, Zurich, Switzerland; b Institute of Food, Nutrition and Health, ETH Zurich, Zurich, Switzerland; c Division of Food Microbial Systems, Microbiological Safety of Foods of Animal Origin Group, Agroscope, Bern, Switzerland; d Biosafety Laboratory, State Laboratory Basel-City, Basel, Switzerland

## Abstract

We present the complete genome sequence of *Escherichia coli* ABWA45, a 16S rRNA methyltransferase-producing wastewater isolate. Assembly and annotation resulted in a 5,094,639-bp circular chromosome and four closed plasmids of 145,220 bp, 113,793 bp, 57,232 bp, and 47,900 bp in size. Furthermore, a small open plasmid (7,537 bp in size) was assembled.

## GENOME ANNOUNCEMENT

The World Health Organization (WHO) defines antimicrobial resistance as one of the major threats to the health and welfare of both humans and animals (1). Aminoglycosides are categorized as critically important antimicrobial agents (2) and are often used in combination with β-lactams for synergistic effects (3). The emergence of plasmid-encoded 16S rRNA methyltransferases in Gram-negative bacteria, in particular in carbapenemase producers, is of great concern since these genes confer high-level resistance against a wide variety of aminoglycosides, including gentamicin, tobramycin, and amikacin (4).

Here, we describe the genome of a 16S rRNA methyltransferase-producing *Escherichia coli* isolate collected from wastewater near Basel, Switzerland, in January 2016 (5). Whole-genome sequencing was performed at the Functional Genomics Center Zurich (FGCZ) using Pacific Biosciences (PacBio) single-molecule real-time (SMRT) technology RS2 reads (C4/P6 chemistry). The reads were *de novo* assembled using SMRTAnalysis 2.3 with the HGAP3 protocol, and sequences were annotated using the NCBI Prokaryotic Genome Annotation Pipeline (6). The genome was assessed using tools (see http://www.genomicepidemiology.org/) that included the MLST-1.8 server (7), ResFinder 2.1 (8), and PlasmidFinder 1.3 (9) to identify sequence type (ST), acquired resistance genes, and plasmid incompatibility types.

*E. coli* ABWA45 belongs to ST635 and is a member of the phylogenetic group A, representing a commensal *E. coli* strain. The closed chromosome is 5,094,639 bp in size with an overall GC content of 50.9%. In addition, the assembly resulted in five plasmids, of which four showed a closed sequence. Only one plasmid, pABWA45_3, which is 57,232 bp in size and has a GC content of 50.1%, encodes antimicrobial resistance determinants: the 16S rRNA methyltransferase gene *rmtB* and the β-lactamase gene *bla*_TEM-1b_. This plasmid consists of a typical IncN backbone structure, and the two resistance genes are flanked by two IS*26* elements in close proximity to a Tn*2* transposase gene. pABWA45_1 is 145,220 bp in size (GC content, 52.9%) and belongs to the IncF incompatibility group. Interestingly, this plasmid revealed no good hits to any other plasmid compared to the NCBI database (closest related plasmid, pKPN-a41 [GenBank accession number CP007735]; query coverage, 24%; identity, 99%). pABWA45_2 is a 113,793-bp nontypeable plasmid (GC content, 54.6%) and resembles a *Klebsiella oxytoca* plasmid, pKPC_UV02 (accession number CP017929; 83% query coverage, 99% identity), but is missing the KPC-encoding Tn*4401*. pABWA45_4 (47,900 bp; GC content, 46.6%) could not be assigned to any incompatibility group by PlasmidFinder but shows substantial homologies to the backbone structure of pBK31567 (accession number JX193302), which belongs to the IncX5 group (10). In contrast to pBK31567, pABWA45_4 does not carry any antimicrobial resistance determinants. pABWA45_5 is a small open plasmid (7,537 bp; GC content, 57.7%) with a closest hit toward pSYM9 (accession number KM107845; 72% query coverage, 96% identity) and carries only putative genes with unknown functions.

Of note, *E. coli* isolates belonging to ST635 have been associated with the production of either New Delhi metallo-β-lactamases or extended-spectrum β-lactamases (11, 12). These findings suggest that ST635 might play an important role in the spread of many different important resistance determinants.

### Accession number(s).

Sequence and annotation data of the genome have been deposited in GenBank under accession numbers CP022154 (chromosome), CP022155 (pABWA45_1), CP022156 (pABWA45_2), CP022157 (pABWA45_3), CP022158 (pABWA45_4), and CP022159 (pABWA45_5). This is the first version of this genome.
